# Late gadolinium enhancement score (LGE-Score) for prediction of extensive late gadolinium enhancement in hypertrophic cardiomyopathy

**DOI:** 10.1186/1532-429X-17-S1-Q59

**Published:** 2015-02-03

**Authors:** Raymond H Chan, Barry J Maron, Iacopo Olivotto, Gabriele Egidy Assenza, Tammy S Haas, John R Lesser, Christiane Gruner, Andrew Crean, Harry Rakowski, James E Udelson, Ethan J Rowin, Massimo Lombardi, Franco Cecchi, Benedetta Tomberli, Paolo Spirito, Francesco Formisano, Elena Biagini, Claudio Rapezzi, Carlo Nicola De Cecco, Camillo Autore, Susie N Hong, Michael C Gibson, Warren J Manning, Evan Appelbaum, Martin Maron

**Affiliations:** 1 Toronto General Hospital, Toronto, ON, Canada; 2 Minneapolis Heart Institute Foundation, Minneapolis, MN, USA; 3 Azienda Ospedaliera Universitaria Careggi, Florence, Italy; 4 Tufts Medical Center, Boston, MA, USA; 5 Beth Israel Deaconess Medical Center, Boston, MA, USA

## Background

Extensive fibrosis detected by late gadolinium enhancement (LGE) in contrast enhanced cardiac magnetic resonance (CMR) has recently been identified as a prognostic marker for adverse events in hypertrophic cardiomyopathy (HCM) patients. However, use of CMR in all patients with HCM may be limited by cost and availability. We therefore sought to develop a score based on clinical and imaging variables to predict the probability of extensive LGE, defined by ≥15% of left ventricular (LV) myocardium with LGE.

## Methods

We assessed the relation between clinical and imaging variables with extensive LGE in an international multicenter cohort of 1293 HCM patients. We used logistic regression to construct integer risk weights for independent variables which predict the presence of extensive LGE. These weights were summed for each patient to create a score (LGE-Score) to predict the probability of extensive LGE.

## Results

Extensive LGE was found in 109 of 1293 patients (8.4%). There were 5 independent predictors for extensive LGE: LV ejection fraction (LVEF) (p<0.0001), history of non-sustained ventricular tachycardia (NSVT) (p<0.0001), history of atrial fibrillation (p=0.02), maximal wall thickness (p=0.0001), and significant resting LV outflow tract gradient (LVOT) >30mmHg (p=0.02). Their associated additive risk-weights in the LGE-Score (in parentheses) were as follows: LVEF ≥70%(0), 50-70% (+4 points), <50% (+8 points); history of NSVT (+3 points); history of atrial fibrillation (+2 points); maximal wall thickness<16.25mm (0), 16.25-19.2mm (+1 points), 19.2-22.9mm (+2 points), ≥22.9mm (+3 points); significant resting LVOT gradient (-2 points). The model has an area under receiver operator curve of 0.794. Using a cutoff LGE-Score of ≤+4 points, the negative predictive value for extensive LGE was 98%.

## Conclusions

Extensive LGE (myocardial fibrosis/scarring) identifies HCM patients at significantly increased risk for sudden death events, however CMR is not available to all patients. For those who are unable to undergo CMR imaging, this novel predictive score can be used to identify those HCM patients who are highly unlikely to have extensive myocardial scarring.

## Funding

Nil.

**Figure 1:**
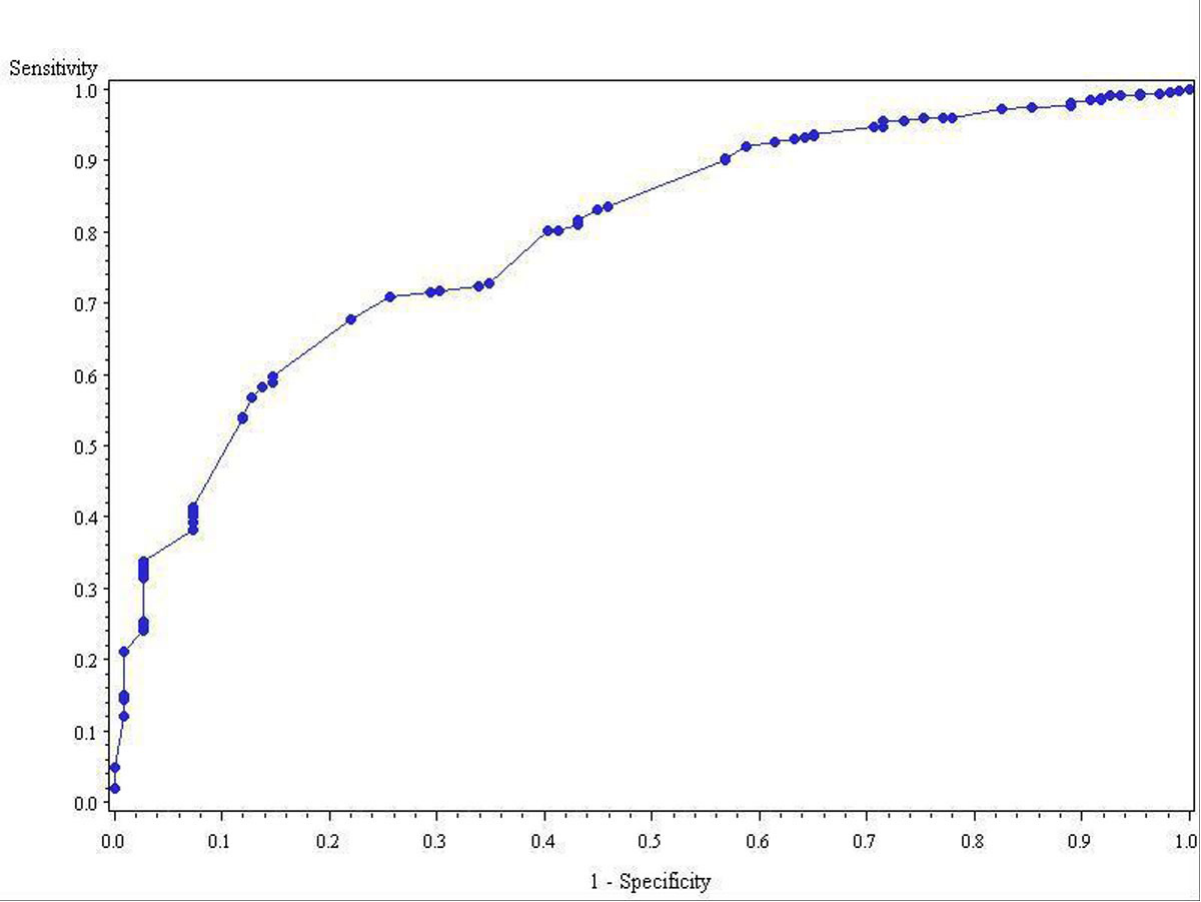
ROC curve for LGE-Score.

